# Assessing Supraspinatus Tendon Elasticity at Different Locations and Loading Conditions Using Ultrasound Shear-Wave Elastography in Young Healthy Population

**DOI:** 10.3390/diagnostics15091132

**Published:** 2025-04-29

**Authors:** Arash Azhideh, Peyman Mirghaderi, Sara Haseli, William D. Lack, Karen C. Takatani, Liisa C. Hammer, Kevin F. Malik, Hannah Tifft, Kyle Griffith, Majid Chalian

**Affiliations:** 1 Division of Musculoskeletal Imaging and Intervention, Department of Radiology, University of Washington, Seattle, WA 98105, USA; azhideh@uw.edu (A.A.);; 2 Department of Orthopaedics and Sports Medicine, University of Washington, Seattle, WA 98105, USA; 3 Boeing Research & Technology, The Boeing Company, Seattle, WA 98108, USA; 4 Department of Orthopaedic Surgery, University of Southern California, Los Angeles, CA 90007, USA

**Keywords:** elastography, shear wave, supraspinatus tendon, elasticity, mechanical properties

## Abstract

**Objective**: This prospective study aims to investigate the feasibility of Shear-Wave Elastography (SWE) for assessing the mechanical properties of the normal supraspinatus tendon and describing the elastographic features of the supraspinatus tendon under different loading conditions and positions. **Methods**: Twenty healthy volunteers (10 males and 10 females, aged 18–25 years) were examined by SWE using an 18-4 MHz linear array transducer. The elasticity of the supraspinatus tendon of the dominant hand was measured at three distinct locations: the insertion, middle, and myotendinous junction of the tendon. These measurements were taken under various conditions, including non-loading and the application of 5, 10, and 20 lb forces at five different positions. **Results**: The average elasticity was 69.2 ± 26.5 kilopascals across all positions and forces, with the middle part of the tendon exhibiting the highest elasticity (72.6 ± 6.2 kPa). An ascending trend in elasticity was observed by increasing the applied load, and the highest elasticity was observed with a 20 lb load. Determining the normal elasticity of the tendon is an important clinical implication, as understanding what is normal is essential for identifying pathological conditions. **Conclusions**: SWE is a feasible and promising technology for the collection of data on how the supraspinatus tendon behaves under loading conditions. There is a need for further study to better understand tendon response to activity and resultant injuries.

## 1. Introduction

Shoulder pain affects approximately 15% to 30% of the general population, with its prevalence rising with age [1]. Rotator cuff tendinopathy is the leading cause of the majority of cases [2]. Among rotator cuff tendinopathies, the supraspinatus tendon is the most affected due to its unique anatomical position between the acromion process and the humeral head [3,4]. Among rotator cuff tendinopathies, the supraspinatus tendon is the most frequently affected due to its anatomical position beneath the acromion, where it traverses the narrow subacromial space. During shoulder movements, particularly abduction and forward flexion beyond 30 degrees, the supraspinatus tendon can be compressed between the acromion and the greater tuberosity of the humerus, predisposing it to impingement and tendinopathy [5]. Among the imaging modalities, ultrasonography (US) stands out as a cost-effective, noninvasive, and accessible option [6,7]. However, conventional US may encounter challenges in accurately diagnosing tendinopathies, especially in the mild and early stages, as the echogenicity of an abnormal tendon might closely resemble that of a healthy one [3,7,8].

The scope of ultrasound elastography (USE) extends to various musculoskeletal tissues, encompassing tendons, muscles, nerves, ligaments, and cartilage [9]. Four main techniques of USE were introduced, including compression or static strain elastography, transient or pulsed elastography, supersonic shear imaging, and shear-wave elastography (SWE) [10]. SWE is a relatively operator-independent and reproducible method offering a quantitative measurement of tissue elasticity in kPa or velocity in centimeters/millimeters per second [9,11]. Although prior studies have investigated SWE in musculoskeletal tissues, most have focused on static conditions or pathological states. The existing literature lacks a systematic evaluation of how supraspinatus tendon elasticity varies under different loading conditions and positional changes. Understanding these variations is essential for establishing normal reference values, enabling the more accurate identification of early pathologic changes. Studies have reported a diminished shear-wave propagation velocity in tendinopathies compared to the resilient stiffness observed in normal tendons [12,13]. This implies that elastography may be helpful in the early diagnosis of tendinopathy before the onset of symptoms due to changes in the material properties of the tendon. However, the lack of information in the literature is a quantitative measure of SWE in healthy individuals.

The purpose of the present study is to investigate the feasibility of SWE to assess the mechanical elastic properties of the normal supraspinatus tendon and to describe the elastographic features of the normal supraspinatus tendon under different loadings.

## 2. Methods and Materials

Our hospital’s institutional review board approved this Health Insurance Portability and Accountability Act (HIPAA) compliant prospective study. All subjects provided written informed consent, which was approved by the local institutional review board (dated 23 September 2021, IRB ID: STUDY00013510).

### 2.1. Study Population

This prospective study included a cohort of 20 healthy volunteers with a mean age of 20 ± 2.4 years (ranging from 18 to 25) and a body mass index of 24.4 ± 5 kg/m^2^ (ranging from 18.6 to 37.1) with no history of shoulder trauma, operative intervention, or known musculoskeletal disorders. The mean age was 20.8 years for men and 19.2 years for women, while the mean BMI was 22.7 kg/m^2^ for men and 26.1 kg/m^2^ for women. All participants had no history of shoulder trauma, surgical intervention, or known musculoskeletal disorders.

Participants with cognitive impairments or language barriers impeding their comprehension of the study’s nature or inhibiting their capacity to provide informed consent were excluded. A comprehensive physical examination of the shoulder consisting of an active and passive range of motion assessment was conducted for all participants by a specialist, and they were required to complete the Disabilities of the Arm, Shoulder, and Hand (DASH) questionnaire (Supplementary Materials), confirming the absence of clinically notable shoulder pathology and discomfort. Age, sex, and body mass index were recorded during the study.

To ensure the assessment of elastography in normal supraspinatus tendons, all volunteers underwent an initial conventional US scan to confirm the normal tendon echogenicity. All of the volunteers had normal activity levels, and none of them were athletic individuals. Tendons with evidence of calcific tendinitis, tendinosis, or even small partial tears, which can occur in asymptomatic individuals, were excluded [14].

### 2.2. Study Design

The US examination of all volunteers, including B-mode and SWE, was performed over one month (November 2021) with the same ultrasound device (Philips EPIQ 7 ultrasound machine, Philips, Amsterdam, The Netherlands) and transducer used (Philips eL 18-4 MHz broadband linear array transducer, Philips, Amsterdam, The Netherlands). The US examinations were performed by a musculoskeletal fellowship-trained radiologist with more than 6 years of clinical experience and expertise in MSK US. The radiologist performing SWE assessments was blinded to the participant data to minimize potential bias in image acquisition and interpretation. The evaluation began with the volunteer seated in a chair, allowing the shoulder to hang naturally alongside the body in a relaxed position. All assessments were conducted on the dominant hand of each participant to maintain uniformity. To obtain the optimal image acquisition, ultrasound gel was applied to the skin’s surface for light contact with the probe and to avoid compression. Once the key section of the supraspinatus tendon was targeted through a B-mode ultrasound assessment, the elastography assessment was initiated.

**Figure 1:**
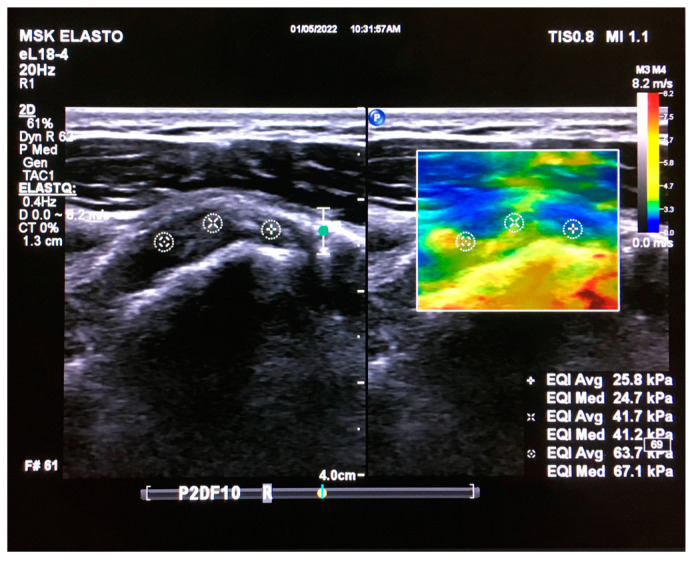
Coronal real-time ultrasound elastography image of the supraspinatus tendon in a healthy young adult. B-mode ultrasound image (**left**) and ultrasound elastogram (**right**). The elasticity was measured at the proximal (+), middle (×), and myotendinous (*) parts of the tendon. A circular ROI with a diameter of 5 mm was manually applied to measure the tendon’s elasticity. Each measurement included the average and median of the elasticity of this circular ROI of the tendon in the rectangular ROI (which varied in size), focusing on the central part of the tendon. The image was obtained from the University of Washington Medical Imaging database with the required permissions.

The conventional B-mode ultrasound image was displayed on the left side of the screen adjacent to the color-coded real-time elastogram on the right side of the screen. The transparency of the color was optimally adjusted to allow the underlying grayscale image to be visible through the superimposed color map. The color code displayed the relative stiffness of the tissues within the ROI, which ranged from blue (soft) to red (stiff). Green and yellow indicated medium elasticity (Figure 1).

**Figure 2:**
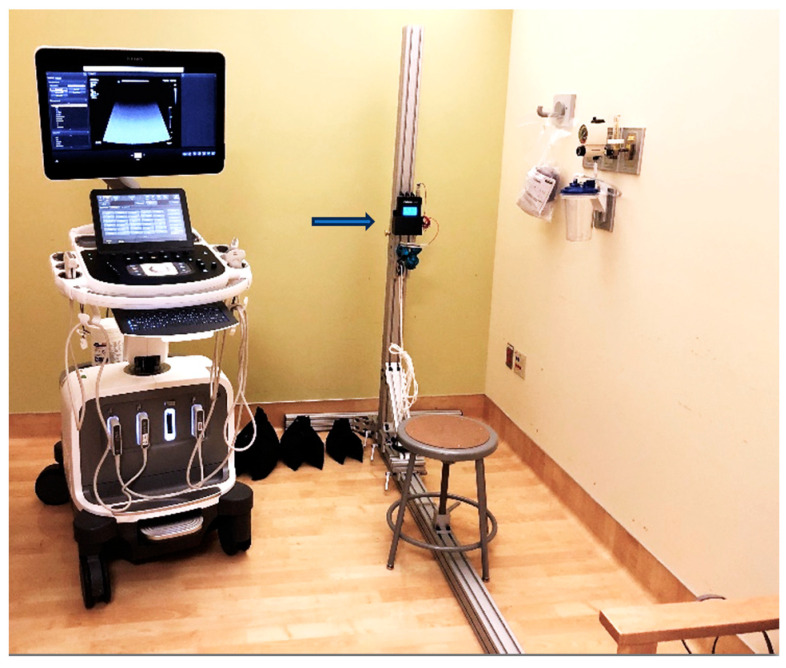
Custom-designed apparatus for applying loads at different upper extremity positions. The aluminum vertical post allows the height of the pulley to be adjusted to ensure the force vector is in line with the lower arm, and a load cell with a digital display (arrow) was used to measure the applied forces.

For the SWE evaluation, we held the probe on the tendon for 3–5 s, allowing the elastography map to stabilize. Subsequently, the acquired images were stored as cine-loops, from which the optimal image for the measurements was chosen. Our measurement process included assessing elasticity at the insertion, middle, and myotendinous junctions. A circular ROI with a diameter of 5 mm was manually applied on the center of the tendon to measure the average and median elasticity. The resultant images with the three measurements were captured as cine-loops. The initial elasticity assessment was carried out without applying any external force. Subsequently, participants were asked to carry 5, 10, and 20 lb loads to evaluate elasticity under varied stress conditions. An apparatus with a digital display for the load was designed with an adjustment mechanism to align a pulley and free-weight system with the height of the participant’s elbow and the direction of force with each participant’s arm, thereby ensuring the direction of force was in line with the participant’s forearm. We employed sandbags to exert forces in specific positions (Figure 2).

**Figure 3:**
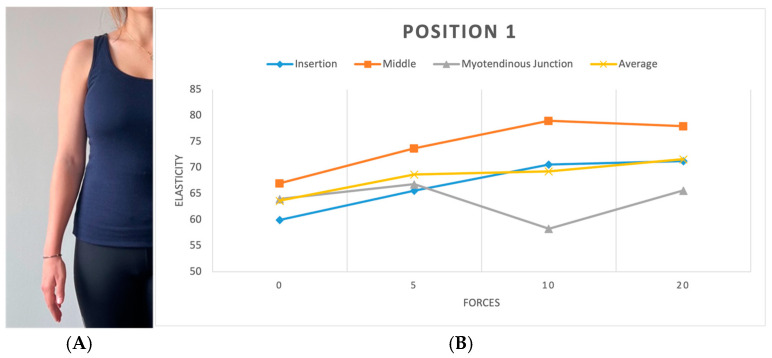
(**A**) Position 1. (**B**) Plot showing elasticity across tendon regions with varying force in position 1. Elasticity is reported in kPa, while forces are measured in lbs.

**Figure 4:**
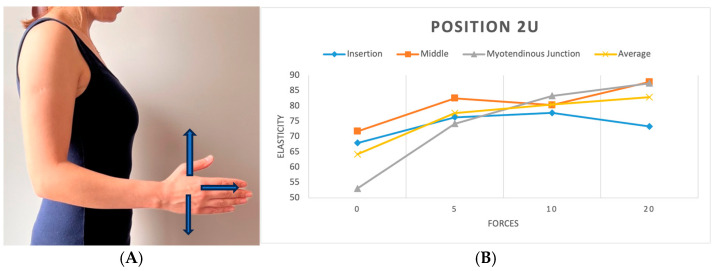
(**A**) Position 2 in three directions (upward, downward, and forward). (**B**) Plot showing elasticity across tendon regions with varying forces in position 2 (upward).

**Figure 5:**
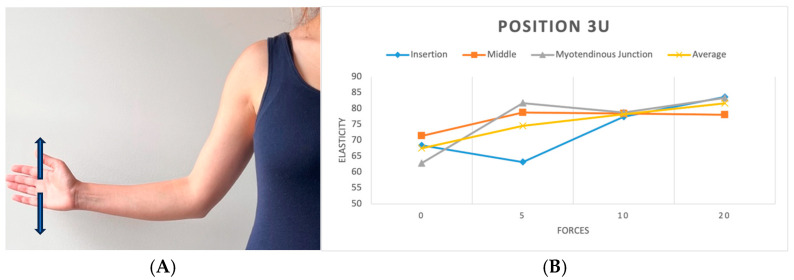
(**A**) Position 3 in two directions (upward and downward). (**B**) Plot showing elasticity across tendon regions with varying force in position 3 (upward).

**Figure 6:**
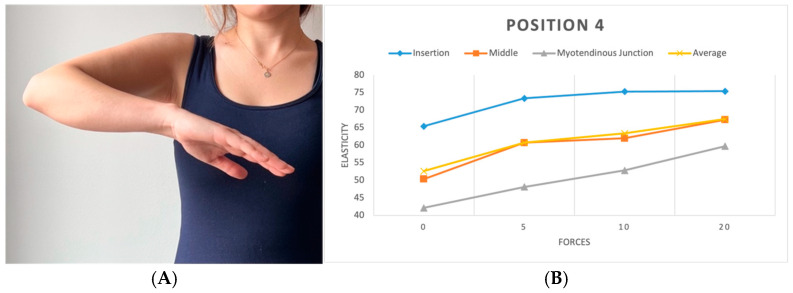
(**A**) Position 4. (**B**) Plots showing elasticity across tendon regions with varying force in position 4.

**Figure 7:**
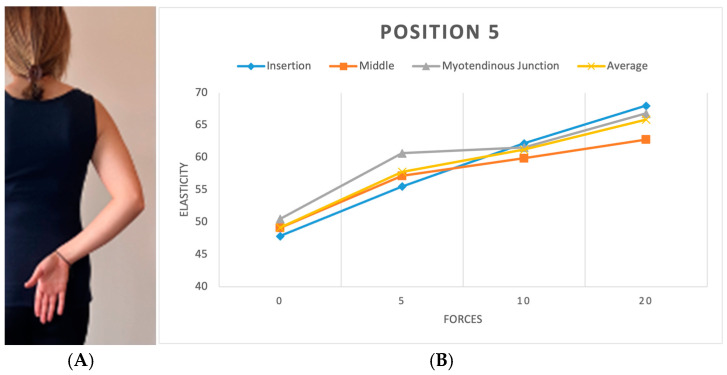
(**A**) Position 5. (**B**) Plots showing elasticity across tendon regions with varying force in position 5.

We replicated this procedure across five distinct positions. In the initial position (P1), the shoulder hung freely alongside the body in a relaxed stance (Figure 3). The second position (P2) was the same as the first one, with a 90-degree flexed elbow, with each force applied in three different directions: upward, downward, and forward (Figure 4). Transitioning to the third position (P3), the shoulder assumed an externally rotated orientation while maintaining a 90-degree elbow flexion. The measurements at the P3 position were duplicated across the upward and downward directions (Figure 5). For the fourth position (P4), the participants were asked to put their hands in front of their chest, which encompassed shoulder flexion, adduction, and internal rotation with the elbow flexed at 90 degrees (Figure 6). For the last position (P5), participants were asked to place their hands in a modified crass position (where the dorsum of the hand is placed behind the back/lumbar region); the internal rotation of the humerus is replicated (see Figure 7). For both P2 and P3 positions, elasticity without force was gauged in the neutral direction, whereas elasticities under 5, 10, and 20 lb loads were quantified in assorted directions. Each participant underwent 29 elasticity measurements (Table 1), with each measurement encompassing the average and median values derived from the insertion, middle, and myotendinous junction segments of the supraspinatus tendon.

### 2.3. Statistical Analysis

The mean elasticity value for each subject was calculated for all variables of interest, including force (0, 5, 10, 20 lb), position (USP1–USP5), direction (upward, downward, forward, neutral), and anatomical location along the tendon (insertion, middle, and myotendinous junction). For conditions with repeated measurements, the average across those measurements was used. To evaluate the overall effects of force and position on tendon elasticity, a two-way analysis of variance (ANOVA) was conducted. Post hoc analysis using Tukey’s Honestly Significant Difference (HSD) test was applied to compare pairwise differences between the levels of each factor. In cases where the two-way ANOVA identified condition-level differences, we performed additional one-way ANOVA to explore the variation in tendon elasticity between positions under the same loading condition and between load levels within the same position. These one-way ANOVAs were also followed by Tukey’s HSD post hoc tests to identify significant pairwise comparisons. Pearson’s correlation coefficient was calculated to explore the linear relationship between increasing load and tendon elasticity in different tendon regions. The significance level of *p* < 0.05 was considered statistically significant for all analyses. All statistical computations were performed using SPSS (IBM SPSS Statistics for Windows, Version 27.0, Armonk, NY, USA: IBM Corp).

## 3. Results

A cohort of 20 healthy volunteers with a mean age of 20 ± 2.4 years (ranging from 18 to 25) and a body mass index of 24.4 ± 5 kg/m^2^ (ranging from 18.6 to 37.1) was recruited. This group was evenly distributed between males and females, comprising 50% females and 50% males. In one instance, a participant experienced discomfort and could not tolerate the 20 lb force during the third ultrasound position in the upward direction. The mean elasticity of all parts of supraspinatus tendons at different positions and directions with various forces was 69.2 ± 26.5 kPa. The mean of this elasticity was 65.8 ± 8 kPa at insertion, 72.6 ± 6.2 kPa in the middle part, and 66.5 ± 9.8 kPa in the myotendinous junction part of the supraspinatus tendon.

### 3.1. Tendon Elasticity at Different Forces

**Figure 8:**
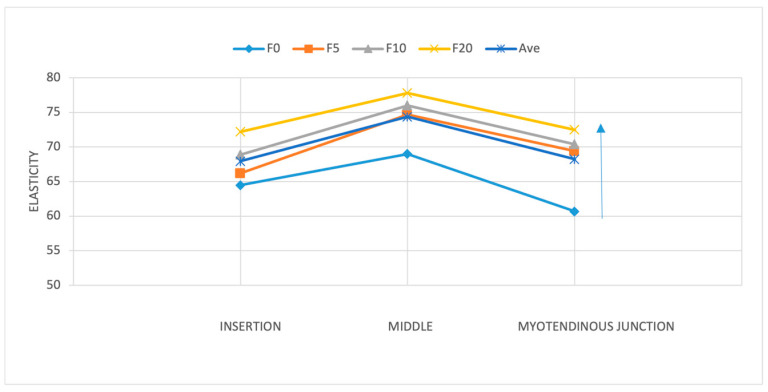
Supraspinatus tendon elasticity across the three tendon regions (insertion, middle, and myotendinous junction) at position 2 under increasing applied loads (0, 5, 10, and 20 lb). The middle portion exhibited the highest elasticity values across all loading conditions. The arrow on the right shows the increase in load.

The analysis revealed significant differences in tendon elasticity when differing forces were applied using the ANOVA test (*p* < 0.01) across all positions and parts of the tendon. Post hoc analysis showed a rise in elasticity when the applied load increased. Although specific load level differences did not reach significance, the presence of any load significantly impacted elasticity compared to no load, particularly at the middle and myotendinous junctions of the tendon. The most notable change in elasticity was found when applying no load compared to the 20 lb load. [9.4 kPa (CI 95%; 4.2, 14.7) *p* < 0.001] (Figure 8). Furthermore, a position-specific analysis was executed to determine instances where increasing the load led to significant changes in elasticity. While an overall correlation of increasing elasticity with increasing force was observed across all positions, statistical significance was limited to position 2 in two directions, both at the myotendinous junction of the tendon (*p* < 0.001). In the upward direction, position 2 demonstrated significance between no load and the 5, 10, and 20 lb loads (Figure 4). A less significant effect was seen in the forward (pushing) direction, with demonstrated significance between no load and the 10 and 20 lb loads.

### 3.2. Change in Elasticity Along the Tendon Anatomy

**Figure 9:**
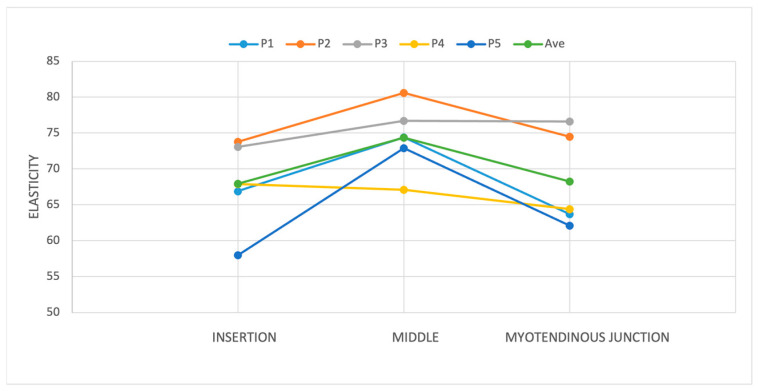
The difference in elasticity was significant between the insertion and middle sections and between the myotendinous junction and middle part, with the highest elasticity observed at the tendon’s middle point. The evaluation of individual positions showed that the difference in elasticity in different parts of the tendon was significant only in positions 1 and 5 (*p* < 0.05).

We investigated whether there was a significant difference between elasticity across three tendon regions: at the insertion, at the middle, and at the myotendinous junction. Our findings highlighted region-specific differences within the tendon. Notably, the middle portion had the highest elasticity compared to the insertion and myotendinous junctions (*p* < 0.001). A detailed post hoc analysis showed that the difference in elasticity was significant between the insertion and middle sections [6.4 kPa (95% CI; 5–16.4)] and between the myotendinous junction and middle part [6.1 kPa (95% CI; 2.5–9.7)], with the highest elasticity observed at the tendon’s middle point (Figure 8 and Figure 9). No significant differences in elasticity were revealed between the insertion and myotendinous junction regions of the tendon. The evaluation of individual positions showed that the difference in elasticity at different locations of the tendon was significant only in positions 1 (*p* < 0.05) and 5 (*p* < 0.001).

### 3.3. Change in Elasticity with Extremity Position and Force Direction

To evaluate the effect of arm position on supraspinatus tendon elasticity under each load condition, separate one-way ANOVAs were performed for the 0 lb, 5 lb, 10 lb, and 20 lb loading groups. A significant difference in elasticity across positions was found at 0 lb (F = 5.32, *p* < 0.01), 5 lb (F = 9.11, *p* < 0.01), and 10 lb (F = 3.15, *p* = 0.0259), while no significant differences were observed at 20 lb (F = 1.22, *p* = 0.3026). A post hoc analysis using Tukey’s HSD revealed that at 0 lb, USP1 differed significantly from USP2 (*p* < 0.01), and USP2 differed from USP4 (*p* < 0.01). At 5 lb, multiple significant pairwise differences were observed, including USP1 vs. USP3 (*p* < 0.01), USP2 vs. USP4 (*p* < 0.01), and USP3 vs. USP4 (*p* < 0.01). At 10 lb, only the comparison between USP1 and USP4 reached statistical significance (*p* = 0.0257). These results suggest that the arm position plays a significant role in tendon elasticity, particularly under lower and moderate loading conditions.

To further assess the relationship between the applied load and tendon stiffness, we performed Pearson’s correlation analysis across different anatomical regions of the supraspinatus tendon. The analysis revealed a weak but statistically significant positive correlation in the insertional region (USP1) (r = 0.157, *p* = 0.015), indicating that elasticity tends to increase as the external load increases. A borderline significant correlation was also observed in the distal region (USP5) (r = 0.120, *p* = 0.064). No significant correlations were found in the middle and deep tendon regions (USP2, USP3, USP4), potentially due to the limited variability or constant elasticity values in those areas.

Although the middle portion of the tendon (USP4) exhibited the highest overall elasticity values across the loading conditions, it did not demonstrate a statistically significant correlation with the increasing load (r = −0.017, *p* = 0.788), and the mean change in elasticity from 0 lb to 20 lb was minimal (65.87 kPa to 64.04 kPa; Δ = −1.84 kPa). In contrast, the insertional region (USP1) showed a more pronounced increase in elasticity with loading (60.97 kPa to 72.23 kPa; Δ = +11.26 kPa), followed by the distal region (USP5) (57.96 kPa to 67.08 kPa; Δ = +9.13 kPa). These findings suggest that while the middle tendon region may have the highest baseline stiffness, the insertion and distal regions are more mechanically responsive to progressive loading.

## 4. Discussion

This study highlights significant variations in the elasticity of the supraspinatus tendon across different anatomical regions, loading conditions, and positions. The middle part of the tendon demonstrated the highest elasticity (72.6 ± 6.2 kPa) compared to the insertional (65.8 ± 8 kPa) and myotendinous junction (66.5 ± 9.8 kPa) regions. Tendon elasticity increased consistently with higher applied loads, with the most notable differences observed when transitioning from a resting state to the maximum load of 20 lbs. Position-specific differences were also evident, particularly in positions where the load direction and tendon orientation played a significant role in altering elasticity. These findings emphasize the non-linear mechanical behavior of the tendon and the importance of analyzing elasticity in a region-specific and load-dependent manner, providing valuable insights into normal tendon behavior and its potential applications in clinical practice.

The elasticity of different regions of the tendon is consistent with prior studies, which suggest that variations in tendon microstructure, collagen alignment, and biomechanical properties contribute to elasticity differences along the tendon. For instance, Arda et al. measured supraspinatus tendon elasticity in healthy volunteers using SWE and reported a mean value of 74.4 ± 45.7 kPa in a standard crass position [15]. While their measurements are slightly higher than our mean values, the variation could be attributed to the differences in methodology, including the position of the arm, the specific SWE equipment used, and inter-study variability in participant demographics [15].

Our findings showed an ascending trend in elasticity with increasing applied loads, with the most significant change observed when progressing from a no-load state to 20 lbs. This load-dependent behavior is supported by studies on other tendons, such as the Achilles tendon, where increased loads have been shown to correlate with higher elasticity and stiffness values [16,17,18]. For example, Leong et al. reported elasticity values for the upper trapezius muscle at rest (17.11 ± 5.82 kPa) and during active arm abduction (26.56 ± 12.32 kPa), demonstrating a load-induced increase in elasticity [19]. Similarly, Hou et al. documented higher shear-wave velocity (SWV) values in healthy supraspinatus tendons (8–9 m/s) compared to pathological tendons (5–7 m/s), reflecting the mechanical resilience of normal tendon tissue under stress [20]. Our study extends these findings by quantifying the elasticity of the supraspinatus tendon under progressive loading conditions.

Position-dependent variations in elasticity further emphasize the importance of standardizing tendon assessments. In our study, position 2, a neutral position, showed significant changes in elasticity with incremental loads, particularly in the upward direction. This observation aligns with studies that highlight the influence of tendon positioning on elasticity measurements [21,22]. For example, Zhang et al. evaluated elasticity in the Achilles tendon following surgical repair and noted that elasticity values correlated with functional outcomes during rehabilitation [23]. These findings underscore the potential for SWE to assess load-specific and position-specific tendon behavior [21,22].

Another key observation in our study was the higher elasticity in the middle portion of the tendon compared to the insertional and myotendinous junction regions. This regional variation has been previously noted in studies such as that by Gadalla et al., who observed SWV differences along the course of the supraspinatus tendon [4]. Their results indicate that the middle portion typically exhibits greater stiffness due to its higher collagen density and alignment. While their SWV values (6–8 m/s in healthy tendons) correspond to elasticity values in the range of 75–100 kPa, differences in SWE devices and methodologies likely account for the slightly higher measurements compared to our findings.

By combining positional and load-specific analyses, our study provides critical insights into tendon behavior under realistic conditions. The consistent increase in elasticity with load application supports the hypothesis that tendons exhibit strain-stiffening behavior, which is an essential biomechanical property that enables tendons to handle increased forces during functional activities. This finding is clinically significant as it highlights the importance of considering loading conditions when assessing tendon health, particularly for identifying early mechanical changes that may predispose tendons to injury [3,4,10,24].

While our study provides valuable insights, several limitations should be considered. SWE measurements were performed by a single operator, and inter-operator variability was not assessed. Future studies should explore larger cohorts, including individuals of different ages, and evaluate inter- and intra-observer reliability to establish robust reference values for clinical use. The sample size of 20 participants was determined based on similar prior studies assessing tendon elasticity using SWE. Although a formal power analysis was not performed, this limitation is acknowledged, and larger sample sizes are recommended to improve statistical power. Additionally, only the supraspinatus tendon of the dominant arm was assessed, limiting our ability to compare bilateral or asymmetrical tendon characteristics. Despite an equal number of male and female participants included in our study, sex/gender-based differences were not analyzed due to the limited sample size. Furthermore, participants’ habitual physical activity levels were not collected, which may influence tendon elasticity and its response to loading. These factors may affect the generalizability of the findings and should be addressed in future research.

## 5. Conclusions

This study assessed the supraspinatus tendon under varying loads and loading directions and in several upper extremity positions to better understand tendon stiffness and elasticity changes. We chose the positions and directions to mimic common positions that the upper extremities occupy in daily activities. We have observed that SWE holds substantial promise as a method for assessing the biomechanical properties of the supraspinatus tendon. Additionally, more comprehensive research comparing elasticity values between normal and pathologic tissues is pivotal to investigating the full potential of SWE in clinical practice and musculoskeletal imaging.

## Figures and Tables

**Table 1:** Applied positions and loads to each participant.

Positions and Directions	Loads (lbs)			
P1	F0	F5	F10	F20
P2 Neutral	F0			
P2 Upward		F5	F10	F20
P2 Downward		F5	F10	F20
P2 Forward		F5	F10	F20
P3 Neutral	F0			
P3 Upward		F5	F10	F20
P3 Downward		F5	F10	F20
P4	F0	F5	F10	F20
P5	F0	F5	F10	F20

F0: no load; F5, F10, F20: 5, 10, and 20 lb loads, respectively. Please note that the measurements for each cell were repeated at the insertion, middle, and myotendinous junction of the tendon, and each measurement obtained the average and median value for elasticity.

## Data Availability

The dataset is available upon request to the editor.

## Supplementary Materials

The following supporting information can be downloaded at https://www.mdpi.com/article/10.3390/diagnostics15091132/s1. Disability of the shoulder.

## Abbreviations

The following abbreviations are used in this manuscript:

SWE	Shear-Wave Elastography
US	Ultrasonography
MRI	Magnetic Resonance Imaging
USE	Ultrasound Elastography
SSI	Supersonic Shear Imaging
ROI	Region of Interest
kPa	Kilopascal
P (1–5)	Position 1, Position 2, Position 3, Position 4, and Position 5 (different experimental positions)
ANOVA	Analysis of Variance
TukeyHSD	Tukey’s Honestly Significant Difference Test
SPSS	Statistical Package for the Social Sciences
SWV	Shear-Wave Velocity
DASH	Disabilities of the Arm, Shoulder, and Hand (Questionnaire)
HIPAA	Health Insurance Portability and Accountability Act
MSK	Musculoskeletal
lb	Pound (weight unit)
CI	Confidence Interval
